# NKG2D-CAR memory T cells target pediatric T-cell acute lymphoblastic leukemia *in vitro* and *in vivo* but fail to eliminate leukemia initiating cells

**DOI:** 10.3389/fimmu.2023.1187665

**Published:** 2023-10-18

**Authors:** Marta Ibáñez-Navarro, Adrián Fernández, Adela Escudero, Gloria Esteso, Carmen Campos-Silva, Miguel Ángel Navarro-Aguadero, Alejandra Leivas, Beatriz Ruz Caracuel, Carlos Rodríguez-Antolín, Alejandra Ortiz, Alfonso Navarro-Zapata, Carmen Mestre-Durán, Manuel Izquierdo, María Balaguer-Pérez, Cristina Ferreras, Joaquín Martínez-López, Mar Valés-Gómez, Antonio Pérez-Martínez, Lucía Fernández

**Affiliations:** ^1^Hematological Malignancies-H12O Lab. Clinical Research Department, Spanish National Cancer Research Centre (CNIO), Madrid, Spain; ^2^Pediatric Oncology Department, Instituto de Genética Médica y Molecular (INGEMM), Hospital Universitario La Paz, Madrid, Spain; ^3^Tumor Immune Activation and Evasion Lab. Immunology and Oncology Department, National Biotechnology Center (CNB), Madrid, Spain; ^4^Hematology Department, Hospital Universitario 12 de Octubre, Madrid, Spain; ^5^Biomarkers and Experimental Therapeutics in Cancer, Hospital La Paz Institute for Health Research-IdiPAZ, Madrid, Spain; ^6^Cancer Epigenetics Laboratory, Genetic Unit, Hospital Universitario La Paz, Madrid, Spain; ^7^Translational Research in Pediatric Oncology, Hematopoietic Transplantation and Cell Therapy, IdiPAZ, Hospital Universitario La Paz, Madrid, Spain; ^8^Instituto de Investigaciones Biomédicas Sols-Morreale (IIBM), Consejo Superior de Investigaciones Científicas, Universidad Autónoma de Madrid (CSIC-UAM), Madrid, Spain; ^9^Pediatric Hemato-Oncology, Hospital Universitario La Paz, Madrid, Spain; ^10^Pediatric Department, Universidad Autónoma de Madrid, Madrid, Spain

**Keywords:** pediatric acute leukemia, T cell lymphoblastic leukemia, NKG2D, NKG2D CAR T cells, leukemia initiating cells

## Abstract

**Introduction:**

Refractory/relapsed pediatric acute leukemia are still clinically challenging and new therapeutic strategies are needed. Interactions between Natural Killer Group 2D (NKG2D) receptor, expressed in cytotoxic immune cells, and its ligands (NKG2DL), which are upregulated in leukemic blasts, are important for anti-leukemia immunosurveillance. Nevertheless, leukemia cells may develop immunoescape strategies as NKG2DL shedding and/or downregulation.

**Methods:**

In this report, we analyzed the anti-leukemia activity of NKG2D chimeric antigen receptor (CAR) redirected memory (CD45RA^-^) T cells *in vitro* and in a murine model of T-cell acute lymphoblastic leukemia (T-ALL). We also explored *in vitro* how soluble NKG2DL (sNKG2DL) affected NKG2D-CAR T cells’ cytotoxicity and the impact of NKG2D-CAR T cells on Jurkat cells gene expression and *in vivo* functionality.

**Results:**

*In vitro*, we found NKG2D-CAR T cells targeted leukemia cells and showed resistance to the immunosuppressive effects exerted by sNKG2DL. *In vivo*, NKG2D-CAR T cells controlled T cell leukemia burden and increased survival of the treated mice but failed to cure the animals. After CAR T cell treatment, Jurkat cells upregulated genes related to proliferation, survival and stemness, and *in vivo*, they exhibited functional properties of leukemia initiating cells.

**Discussion:**

The data here presented suggest, that, in combination with other therapeutic approaches, NKG2D-CAR T cells could be a novel treatment for pediatric T-ALL.

## 1 Introduction

Acute leukemia is the most common cancer in children, and the second leading cause of cancer-related death before 20 years of age (1, 2). Acute lymphoblastic leukemia (ALL) accounts for approximately 80% of leukemia cases in children and 56% of leukemia cases in adolescents, being most of them B-cell immunophenotype (80%). Pediatric patients with T-cell immunophenotype (T-ALL) comprise 10-15% of pediatric ALL (3). With improvements in risk-directed therapy and supportive care, event-free survival (EFS) rates for children with T-ALL now approach 84% and overall survival (OS) is close to 90% (4). However, for refractory/relapsed (r/r) T-ALL few treatment options exist and chances of cure are dismal. In the last years, immunotherapy with T cells redirected with chimeric antigen receptors (CAR) has shown great potential and CD19 CAR-T cells are currently an attractive therapeutic option for pediatric patients with high-risk B-ALL. However, for T-ALL, CAR-T cell approach needs to overcome different hurdles including self-targeting, fratricide and T cell aplasia, caused by the shared expression of target antigens between CAR-T cells, tumor cells and normal T cells (5).

NKG2D receptor is an activating receptor that plays an important role in protecting the host from infections and cancer (6). The expression of ligands for NKG2D receptor (NKG2DL), namely MICA, MICB, and the UL16 binding proteins (ULBP)1-6 has been reported in different tumor types including pediatric AML and ALL (7, 8), making them attractive targets for NKG2D-CAR based therapies. In fact, Driouk et al. have reported NKG2DL expression in T-ALL and AML cells and robust anti-leukemia specific activity of NKG2D-CAR T cells (9). Furthermore, an adult patient suffering from relapsed/refractory AML (r/rAML) showed complete remission (CR) after treatment with autologous NKG2D-CAR T cells (10).

CD45RA^-^ T cell subset comprises most central memory and effector memory T cells (T_CM_ and T_EM_), exerts a memory response to prior pathogens or vaccines and can mediate graft-versus-tumor (GvT) effects without inducing graft-versus-host disease (GvHD) (11–13). These characteristics make from CD45RA^-^ T ideal effector cells to manufacture allogeneic “off-the-self” CAR-T cell products. Our group has already reported an efficient killing of osteosarcoma cells by NKG2D-CAR redirected CD45RA^-^ cells, and developed a protocol to manufacture these CAR-T cells for clinical use (14, 15).

An effective and safe CAR-T cell therapy for r/r non-B cell leukemia is yet to be found. After chemotherapy and even when allogeneic hematopoietic stem cell transplantation (HSCT) is performed, most patients still have poor long-term survival and high relapse rate (16–18). Treatment with CAR-T cells can enhance remission and improve outcomes although some cases quickly relapse (19). In this report, we provide preclinical data to support that NKG2D-CAR redirected CD45RA^-^ T cells (from now on, NKG2D-CAR T cells) could be a novel therapeutic approach for pediatric acute leukemia. NKG2DL were expressed in leukemia cell lines and primary leukemic blasts from pediatric patients. *In vitro*, NKG2D-CAR T cells targeted leukemic blasts being AML and T-ALL blasts the most sensitive to CAR-T cell killing. In a murine model of human T-ALL, NKG2D CAR-T cells delayed leukemia progression and prolonged survival, without treatment related toxicity signs, even at the highest doses. Anti-tumor activity of NKG2D-CAR T cells remained unaltered upon culture with sNKG2DL. However, they failed to completely eliminate the leukemia initiating cells (LICs) compartment, and this occurred despite NKG2DL expression. The data here presented suggest that pediatric patients suffering from T-ALL could benefit from the use of NKG2D-CAR T cells, although other treatment strategies targeting LICs compartment may be needed to completely eradicate leukemia.

## 2 Materials and methods

### 2.1 Patients

Bone marrow (BM) or peripheral blood (PB) samples from pediatric leukemia patients were obtained at the time of diagnosis prior to therapy, at follow-up or in complete remission (CR), as indicated. All patients or their guardians gave their written informed consent in accordance with the Helsinki protocol, and the study was performed according to the guidelines of the local Ethics Committee (PI-3374). BM and peripheral blood mononuclear cells (PBMCs) were obtained by using Ficoll-Hypaque gradient centrifugation and washed twice with PBS before further analysis.

### 2.2 Cell lines and culture

K562, Kasumi, CCRF-CEM, Jurkat, MOLT-3, MV4-11, REH, NALM-6 and RS4-11 cell lines were acquired from ATCC. SEM, ME-1, and TOM-1 were acquired from DSMZ. Jurkat-GFP-Luc cells were gently provided by Professor P. Menéndez, from Josep Carreras Leukemia Research Institute. All cell lines were cultured in RPMI and 10%FBS, except for SEM cell line, which was cultured in IMDM 10%FBS. All cell lines were routinely tested for Mycoplasma by using a DNA-binding dye-based qPCR system as previously described (15).

### 2.3 CAR-expressing lentiviral production, memory T-cell isolation, activation, transduction and expansion

The HL20i4r-MNDantiCD19bbz lentiviral vectors were derived from the clinical vector CL20i4r-EF1a-hgcOPT27 but expressed an NKG2D-CAR. The anti-CD19-4-1BB-CD3z CAR designed by Imai et al. (20) was used as backbone to build the NKG2D-CAR construct. It contained the extracellular domain of NKG2D (designed by Wai-Hang Leung and Wing Leung), the hinge region of CD8a, and the signaling domains of 4-1BB and CD3z. CAR-expressing viral particles pseudotyped with vesicular stomatitis virus G glycoprotein (VSVG) were generated in HEK293T cells by standard transfection protocols using Lipofectamine and concentrated by ultracentrifugation, as previously described (21). Viral titers were consistently in the range of 10^8^ TU/mL. Buffy coats from healthy volunteers were obtained from the Transfusions Centre of the Comunidad de Madrid (CAM) upon institutional review board approval (PI-3374). PBMCs were isolated by Ficoll gradient centrifugation. Isolation and activation of CD45RA^-^ cells were performed as previously described (14). Transduction of CD45RA^-^ cells with lentiviral particles expressing NKG2D-CAR was performed using MOI=2. The cells were harvested 4-6 days later for experiments.

### 2.4 Flow cytometry

NKG2D expression and immunophenotype of CAR T cells, identification of blast subpopulation and NKG2DL expression were determined by flow cytometry (FCM) using either a BD FACSCanto II or LSR Fortessa (both from BD Biosciences)or a Navios flow cytometer (Beckman Coulter). The different fluorochrome-labeled mAbs are summarized in **Supplemental Table ST1**. Blasts were defined as CD45^med^. The specific fluorescence intensity (SFI) was defined as the mean fluorescence intensity (MFI) of the specific staining relative to the MFI of the unstained cells. Positivity for a specific ligand was considered for SFI values ≥2 (22). FCM analysis was performed using FCS Express 7 software (*De Novo* Software) and GraphPad Prism was used for data analysis.

### 2.5 Cytotoxicity assays

Cytotoxicity of NKG2D CAR- T cells against leukemic cells was evaluated at the specified effector-to-target (E:T) ratios by performing 4-hour europium-TDA release assays (PerkinElmer; AD0116) as previously described (14). In some experiments, NKG2D-CAR T cells were incubated with sNKG2DL for 72h before the cytotoxicity assays, or sNKG2DL were added directly to the supernatant of the co-cultures. When primary leukemic blasts were used as targets, samples were obtained at diagnosis and had at least 60% of blasts.

### 2.6 Murine model of human T-ALL

All procedures were approved by the Spanish National Cancer Research Centre (CNIO) Animal Care and Use Committee, by the ethics committee of the Instituto de Salud Carlos III (ISCIII) and the CAM (PROEX173/17). Immunodeficient NOD.Cg-Prkdc^scid^ Il2rg^tm1Wjl^ /SzJ (NSG) mice were bred at the Animal Facility Unit of the CNIO. 5-to-8-week-old mice were engrafted with 1x10^6^ of Jurkat-GFP-luc cells by intravenous (IV) injection through the tail vein at day -3. At day 0, mice were divided into 4 groups: untreated control group receiving IV infusions of PBS (n = 4), mice receiving multiple infusions of 1x10^7^ untransduced memory T cells/mouse (one infusion per week for a total of three weeks) (n = 4), mice receiving a single infusion of 1x10^7^ NKG2D-CAR T cells/mouse (n = 5), mice receiving multiple infusions of NKG2D-CAR T cells divided as follows weekly infusions of 1x10^7^ cells/mouse for three weeks, followed by weekly infusions of 2x10^7^ cells/mouse (n = 5) for three additional weeks (six infusions in total). Mice receiving cells were also administered subcutaneous (SC) injections of IL-2 (250 IU/mouse) every other day for 3 weeks. Tumor burden was monitored weekly by bioluminescence (BLI) of luciferase-expressing cells using Xenogen IVIS 200 Imaging System (Perkin Elmer) 10 minutes later of intraperitoneal administration of 200 µl of D-luciferin 15 mg/ml (Perkin Elmer). BLI images were analyzed using the Living Image 3.0 software (Perkin Elmer). Mice were euthanized when they displayed clinical symptoms of leukemia progression. The persistence of CAR T cells (CD45human^+^GFP^-^NKG2D^+^) and leukemia burden (CD45human^+^GFP^+^) were analyzed by FCM in BM, spleen and PB.

### 2.7 Leukemia-initiating cell assays

To determine T-ALL LICs activity after NKG2D-CAR treatment, Jurkat GFP-luc cells were co-cultured overnight with NKG2D-CAR T cells at an E:T ratio of 1:5. Remaining alive Jurkat-GFP luc cells (Exp-JKT) were then recovered by Fluorescence-activated Cell Sorting (FACS), and engrafted into NSG mice at different cell doses ranging from 1x10^6^ to 10. Same doses of unexposed Jurkat GFP-luc cells (Ctrl-JKT) were engrafted in control mice. Leukemia burden was monitored by BLI measure as explained before. Frequency of mice developing leukemia and survival were analyzed. ELDA software (https://bioinf.wehi.edu.au/software/elda/) was used to calculate the LIC Frequency.

### 2.8 Determination of sNKG2DL in the sera of patients and mice

Serum samples from leukemia patients were collected at different disease time-points, and from T-ALL bearing mice before and after treatment with NKG2D-CAR T cells. The sNKG2DL were quantified by Enzyme Linked Immunosorbent Assay (ELISA). The specific anti-NKG2DL antibodies, NKG2DL recombinant human proteins and HRP antibodies are summarized in **Supplemental Table ST2**. For protein detection, 100ul of ABTS or TMB solution were added and the plates read at 405-492 nm using a SUNRISE microplate reader (Tecan Trading AG).

### 2.9 Effects of sNKG2DL on NKG2D-CAR T cells

NKG2D-CAR T cells were incubated with the specified concentrations of recombinant human proteins of sNKG2DL (ST2) for one week. NKG2D expression was explored by FCM analysis and using a Gallios flow cytometer and Kaluza software (14). The effects of sNKG2DL in the formation of immunological synapses were evaluated by epifluorescence and confocal microscopy, as previously described (23). Briefly, NKG2D-CAR T cells were incubated with 500ng/ml of sMICA for 72 h and co-cultured for 30 minutes with CMAC-labeled Jurkat cells at 1:1 ratio. Cells were then fixed with 4% paraformaldehyde (PFA) and stained with anti-NKG2D, anti-pericentrin to label Microtubule-Organizing Center (MTOC) and phalloidin to localize F-actin. Epifluorescence imaging was performed using a Nikon Eclipse TiE microscope. Confocal imaging was performed using a Leica SP5 confocal microscope (Leica) at 40× magnification. Quantification of digital images was performed using NIS-AR (Nikon) or ImageJ software. To establish the relative ability of the MTOC to polarize toward the immune synapses, MTOC polarization index (MTOC PI) were calculated as explained in **Supplemental Figure 1**. Image analysis data correspond to one experiment, analyzing a minimum of 24 synapses in randomly selected microscopy fields. Unpaired t tests analysis was performed for statistical significance of the results using GraphPad.

### 2.10 Side population analysis and colony forming assays

Jurkat GFP-luc or MOLT-3 cells were incubated overnight with NKG2D-CAR T cells at an E:T ratio of 1:5. Cells were then harvested and left at 1x10^6^cells/ml in RPMI 10%FBS. For those experiments using MOLT-3 cells, NKG2D-CAR T cells were previously labeled with 1µM CFSE CellTrace™ Proliferation kit for FCM (ThermoFisher Scientific, C34570) for 20 min at RT in darkness and then washed. One functional characteristic of LICs is their ability to efflux Hoechst 33342, which reflects enhanced therapy resistance. Upon staining, a side population can be easily identified by FCM. Side population analysis of Jurkat GFP-luc and MOLT-3 cells was performed by FCM as previously described (24). In brief, cells were stained with dye cycle violet (DCV) (5 μM, ThermoFisher Scientific) for 90 min at 37°C in the absence or presence of the ABC transporter inhibitor reserpine (50 μM, Sigma-Aldrich). The gating strategy was based on dead/live cells and doublet discrimination. DCV was excited at 405 nm and its emission fluorescence was detected using 450/50 nm (DCV blue) and 675/20 (DCV red) band pass filter systems. For Jurkat cells, GFP^+^ cells were then sorted for further analysis. Another characteristic of LICs is their clonogenic ability. Colony forming assays were performed as previously described (14) after co-culture of Jurkat or MOLT-3 cells with NKG2D-CAR T cells at the specified ratios for 4h.

### 2.11 Transcriptome and qPCR analysis

Sorted Jurkat-GFP cells were washed in PBS and pelleted in RLT lysis buffer supplemented with 1:1000 β-mercaptoethanol. RNA isolation and sequencing was carried out as previously reported (25). Reads from RNA-seq were analyzed to quantify genes through the RSEM (26) with hg19 as reference for annotation. The differential expression was carried out with edgeR (27)and the statistical cut-off point was set as FDR < 0.05 and logFC >2. Genes were filtered based on a minimum expression of one Counts Per Million (CPM) in more than one sample. Normalization was performed by the TMM method (trimmed mean of M-values) (28). Functional enrichment of differentially expressed genes was carried out in PFAM (29), GOTERM_BP_DIRECT (30) and KEGG REACTOME (31). For qPCR analysis of STAT1, SMAD1, BCL-2, LIF, CD52 and L1CAM genes, MOLT-3 and CCRF-CEM cells were co-cultured overnight at E:T 1:5 with CFSE labeled NKG2D-CAR T cells. At the following day, CFSE negative remaining alive cells were sorted, pelleted in RLT buffer and stored at -80°C. RNA was extracted using RNeasy kit (Qiagen. #74104) following the manufacturer’s instructions. cDNA was obtained using MultiScribe Reverse Transcriptase (Applied Biosystems #4311235). For quantitative real‐time PCR, PowerUP™ SYBR™ Green Master Mix (Applied Biosystems #A25741) was used. ViiA 7 Real-Time PCR System was used to perform the reaction (Applied Biosystems). Values were corrected by GAPDH expression. The 2−ΔΔCt method was applied to calculate the relative gene expression. The primers used for the amplification are specified in **Supplemental Table ST3**.

### 2.12 Statistical analysis

All statistical analyses were performed using Prism software (GraphPad Software). Data are presented as mean ± Standard Error of Mean (SEM). Before any other statistical test, normality of the data set was checked using Kolmogorov-Smirnov test to determine Gaussian distribution. When comparing two groups, paired or unpaired two-tailed Student t-tests were applied. When comparing more than two groups, one-way or two-way analysis of variance (ANOVA) was performed for one or two different variables, respectively. To correlate two variables, Pearson correlation test was performed. The survival curves from the *in vivo* experiments were analyzed by applying the Kaplan-Meier method. P < 0.05 was considered statistically significant.

## 3 Results

### 3.1 NKG2DL are expressed on pediatric leukemia blasts

**Figure 1:**
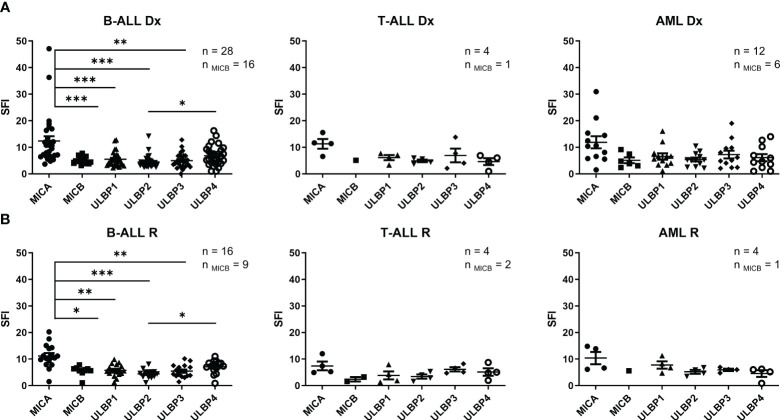
NKG2DL expression in leukemia cells. **(A)** Expression of NKG2DL by FCM of B-ALL, T-ALL and AML pediatric patients blasts at diagnosis. **(B)** Expression of NKG2DL by FCM of B-ALL, T-ALL and AML pediatric patients blasts at relapse. One-way ANOVA was performed for multiple comparisons. (*p ≤ 0.05) (**p ≤0.01), (***p ≤ 0.001).

Leukemia cell lines from myeloid (K562, ME-1, Kasumi-1), B cell (REH, NALM-6, TOM-1, RS4-11), T cell (CEM, Jurkat, MOLT-3) and biphenotypic (MV4-11, SEM) lineages, and 68 primary leukemia samples at diagnosis or relapse (AML=16, B-ALL=44, T-ALL=8) were analyzed for NKG2DL expression by FCM. The SFI detected for individual NKG2DL in the leukemia cell lines are shown in **Table 1**. Representative histograms of NKG2DL analysis of leukemia cell lines from each leukemia entity are shown in **Supplemental Figure SF2**. The expression of NKG2DL in primary leukemic blasts at diagnosis and relapse are shown in **Figure 1A** and in **Figure 1B** respectively. Surface expression of at least one NKG2DL (SFI≥2) was detected in all the studied samples. We also found NKG2DL expression was heterogeneous with no specific patterns associated with leukemia entities.

**Table 1:** NKG2DL expression in leukemia cell lines.

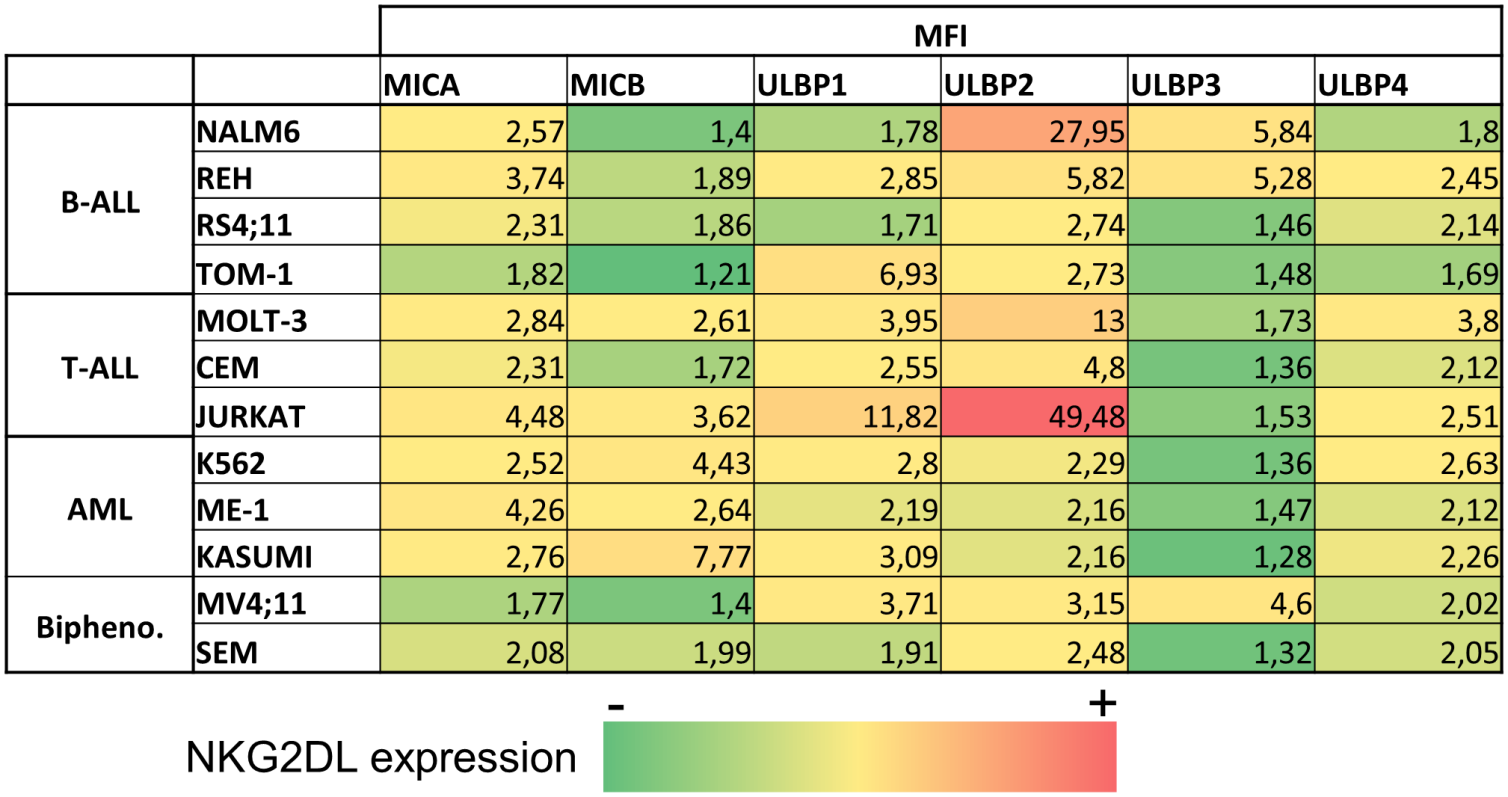	

Numbers represent the average of Specific Fluorescence Intensity obtained in three independent experiments. Positivity of a specific ligand was considered for SFI values ≥2. Color gradient represents lower SFI, between 1-2 in green, low-moderate(2-10) SFI between (yellow) and higher SFI (>10) (red).

Additionally, no significant differences were observed between the expression of NKG2DL at diagnosis and relapse. At diagnosis, MICA showed the highest expression in all leukemia entities, although this difference was statistically significant only in B-ALL samples (**Figure 1A**). Detection of NKG2DL transcripts by quantitative RT-PCR did not always translate into detectable NKG2DL surface expression. (**Supplemental Figure SF3**).

To sum up, these data demonstrate leukemic blasts express NKG2DL and could be a target for NKG2D-CAR therapy.

### 3.2 NKG2D-CAR T cells target leukemia cells *in vitro*

**Figure 2:**
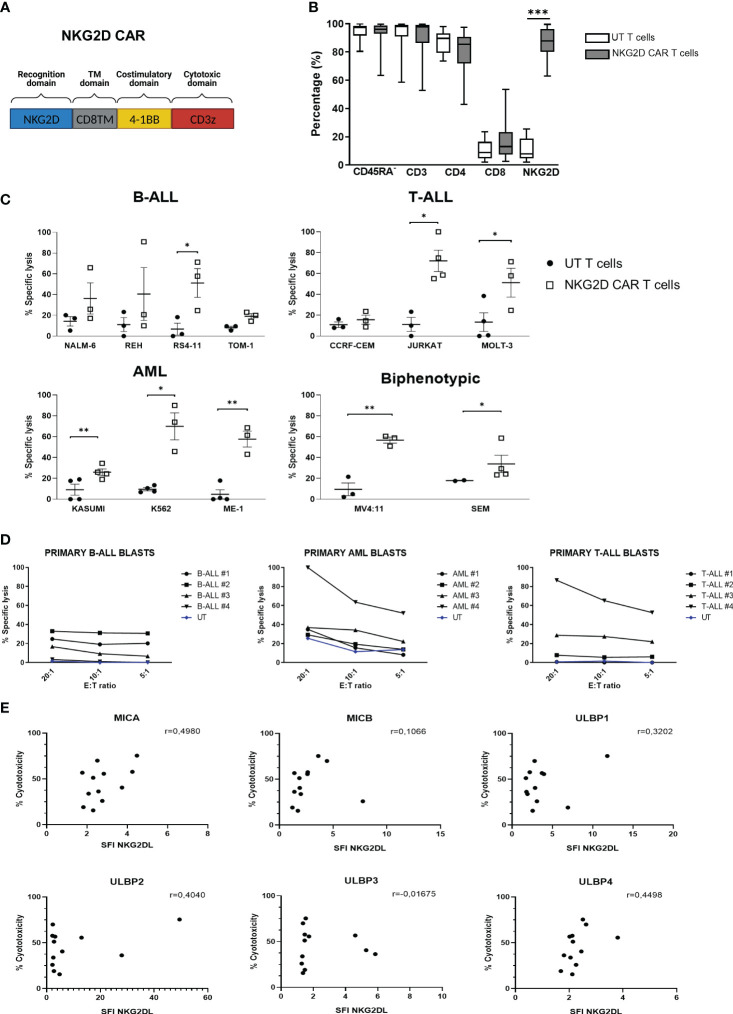
Cytotoxicity *in vitro* of NKG2D-CAR T cells against leukemia cells. **(A)** Schematic representation of NKG2D-CAR and NKG2D CAR expression in CD45RA- cells. The NKG2D-CAR construct contains the extracellular domain of NKG2D, the hinge region of CD8a, and the signaling domains of 4-1BB and CD3z. **(B)** Percentage of CD45RA-, CD3, CD4, CD8 and NKG2D expression in Untransduced (UT) T cells (N=16) and NKG2D CAR T cells (N=18). Nonparametric Mann-Whitney test was used to compare two groups. ***p < 0,001. Data are presented as Mean, maximum and minimum **(C)** Specific lysis by Eu-TDA assays of memory NKG2D-CAR and UT T cells against leukemia cell lines at a 20:1 E:T ratio. Data are presented as single data points and mean ± SEM of independent experiments using different NKG2D CAR T cell donors. For each cell line N=3 except for MOLT-3, Jurkat and SEM cell lines where N=4. **(D)** Specific lysis of memory NKG2D-CAR and UT T cells against primary leukemic blasts at different E:T ratios. Student’s t test was used to compare two groups. *p < 0,05; **p < 0,01; ***p < 0,001; ns, not significant. **(E)** Correlation analysis between NKG2DL expression of leukemia cell lines and their susceptibility to NKG2D-CART cytotoxicity. Parametric Pearson correlation analysis was applied between X and Y values.

Having shown the suitability of using NKG2D-CAR T cells to target acute pediatric leukemia, we next tested the cytotoxicity of NKG2D-CAR memory T cells against leukemia cells *in vitro*. A schema of the NKG2D-CAR construct used for this study is shown in **Figure 2A** The NKG2D-CAR T cells used for the experiments had a median percentage of NKG2D expression of 87.76% ranging from 62.88-99.54%. The percentages of CD45RA^-^, CD3, CD4, CD8 and NKG2D expression in Untransduced (UT) T cells and NKG2D-CAR T cells are shown in **Figure 2B**. NKG2D-CAR T cells showed increased cytotoxic ability compared to untransduced (UT) memory T cells in all the experiments. NKG2D-CAR T cells showed the highest cytotoxicity values against AML and T-ALL cell lines, except for CEM cell line in the T-ALL group, which showed the lowest sensitivity to NKG2D-CAR T cells (**Figure 2C**). The cytotoxicity of NKG2D-CAR T cells against leukemia cell lines at lower E:T ratios is showed in **Supplemental Figure SF4**. When primary blasts were used as targets, AML showed the highest sensitivity followed by T-ALL and B-ALL (**Figure 2D**). The sensitivity of primary blasts to NKG2D CAR T cells lysis showed to be heterogeneous and patient dependent. The percentage of cytotoxicity at a 20:1 E:T ratio ranged from 29.2-100% for AML blasts, from 0.75-86.6% for T-ALL and from 3.3-33% for B-ALL blasts. We next explored if there was correlation between NKG2DL expression and NKG2D-CAR T cells´ cytotoxicity. We considered expression of individual ligands, since NKG2D-CAR may have different binding affinity for different ligands, as it occurs with the endogenous NKG2D receptor (32). Moreover, in addition to their differences in binding affinity, the NKG2DL may have different ability to induce NKG2D-CAR T cells´ activation. We found no correlation between individual NKG2DL expression and sensitivity to NKG2D-CAR T cells (**Figure 2E**). Furthermore, no correlation was found when collective expression of all NKG2DL was considered for the analysis (**Supplemental Figure 5**).

### 3.3 NKG2D-CAR T cells reduce tumor progression in a murine model of T-ALL

**Figure 3:**
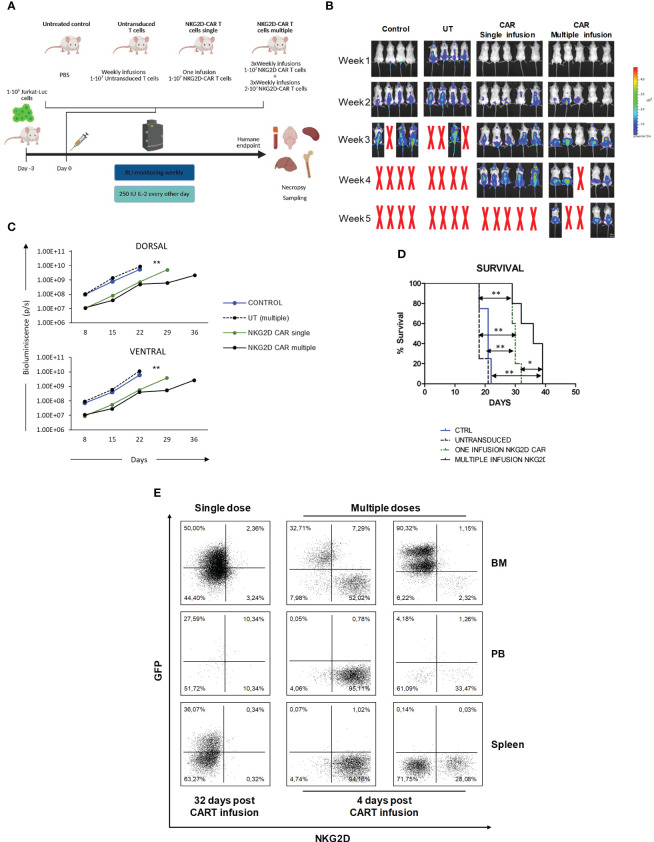
*In vivo* efficacy of NKG2D CAR T cells against Jurkat cells. **(A)** Schema of xenograft model NSG mice (n= 4-5/group) were IV injected with 1x10^6^ Jurkat-GFP-luc cells and then left untreated, treated with untransduced T cells, single or multiple does of NKG2D CAR T cells. **(B)** Representative BLI images of mice engrafted with Jurkat-GFP-luc cells, either left untreated (N=4), treated with multiple doses of UT T cells (N=4), or treated with single (N=5) or multiple doses (N=5) of NKG2D CAR T cells. **(C)** Comparison of ventral and dorsal BLI signals of untreated control mice (blue line), UT (dashed black line), single dose of NKG2D CAR T cells (green line) and multiple doses of NKG2D CAR T cells (bold black line). **(D)** Kaplan–Meier survival curves for control mice (blue line), UT (dashed black line), CAR single (dotted green line) and CAR multiple (bold black line). **(E)** FCM analysis of persistence and homing of NKG2D CAR T cells in the bone marrow (BM), peripheral blood (PB) and spleen. Samples taken from treated mice at day +32 (single dose) and +4 (multiple doses) after last CAR T infusion. NKG2D CAR T cells were detected as GFP^-^/NKG2D CAR^+^.

We next explored the activity of NKG2D-CAR T cells in a murine model of human T-ALL using Jurkat-luc cells. A schema of the T-ALL murine model is shown in **Figure 3A**. Compared with the control groups, treated groups showed a delayed leukemia burden and prolonged survival (**Figures 3B–D**). Based on BLI, both control groups had a similar pattern of leukemia progression and survival. In both treated groups, leukemia progression was controlled until day+22. However, by day 29, those mice treated with an only infusion of NKG2D-CAR T cells, showed increased BLI (**Figure 3C**). Additionally, BLI was detected not only in the bone marrow, but also in the spinal cord and the brain, indicating tumor spreading (**Figure 3B**). In the group receiving multiple infusions, leukemia progression remained controlled until day 29. However, by day 36, BLI signal increased, indicating leukemia progression (**Figure 3C**). FCM analysis revealed that, 32 days after the infusion, a low percentage of NKG2D-CAR T cells (defined as NKG2D^+^GFP^-^) could be found in the PB, the BM and the spleen of those mice receiving an only dose. In those mice receiving multiple doses, a large percentage of CAR T cells was found in all the hematopoietic organs studied, although, it is important to note, that due to the experimental design, the samples were taken only 4 days after the last infusion (**Figure 3E**). Additionally, immunohistochemistry analysis revealed the expression of Granzyme B in the infiltrated CAR-T cells (**Supplemental Figure 6**). All these data taken together indicate that NKG2D-CAR T cells were homing the bone marrow and secreting lytic granules in response to leukemic blasts and controlling leukemia progression and prolonging survival of the treated mice. However, they failed to completely eradicate the leukemia and the animals died from leukemia progression afterwards.

### 3.4 Impact of sNKG2DL in NKG2D-CAR T cells

**Figure 4:**
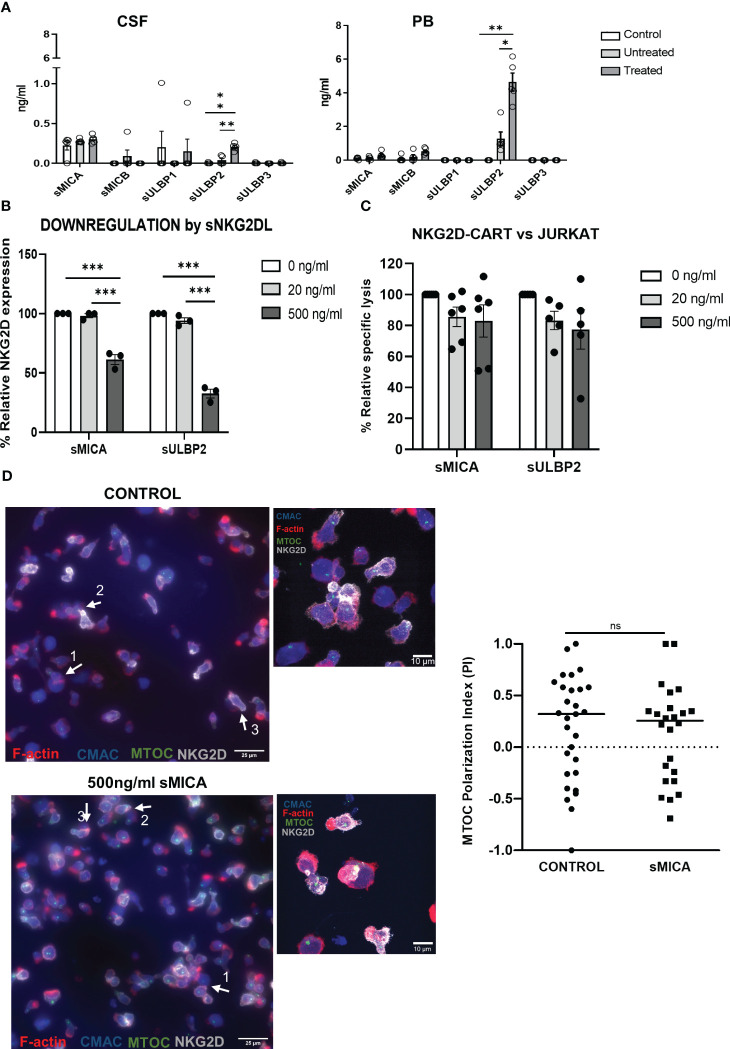
Impact of sNKG2DL in NKG2D-CAR T cells. **(A)** Levels of sNKG2DL measured by ELISA in samples of healthy control NSG mice (N=5), and T-ALL bearing mice either untreated (N=5) or treated with NKG2D CAR-CD45RA^-^ T cells (N=5). Two-way ANOVA following Dunn´s *post-hoc* test was performed. *p < 0,05; **p < 0,01; ***p < 0,001. **(B)** Relative expression of NKG2D of NKG2D-CAR T cells treated with different amounts of sMICA and sULBP2. Three different NKG2D CAR T cell donors were used for the experiments (N=3). Two-way ANOVA following Tukey’s *post-hoc* test was performed. *p < 0,05; **p < 0,01; ***p < 0,001. **(C)** Cytotoxicity of NKG2D CAR T cells against Jurkat cells after treatment for 72h with sMICA and sULBP2. Five different NKG2D CAR T cell donors were used for the experiments (N=5). Two-way ANOVA following Tukey’s *post-hoc* test was performed. *p < 0,05; **p < 0,01; ***p < 0,001. **(D)** Epifluorescence (upper panels) and confocal (lower panels) microscopy images of immune synapses when CMAC-labelled Jurkat cells (blue) were challenged with NKG2D-CAR T cells at 1:1 E:T ratio for 30 minutes. Quantification of MTOC polarization index showed no differences in the formation of immunological synapses after treatment for 72h with sMICA. Unpaired t test was used for comparison between two groups.

To explore if the release of sNKG2DL by leukemic cells could be hindering the anti-tumor ability of NKG2D-CAR T cells in the murine model, we measured the levels of sNKG2DL and their impact on NKG2D-CAR T cells. We found significantly increased levels of sMICB and sULBP2 in diseased mice compared to healthy controls, indicating T-ALL cells released sNKG2DL. Furthermore, treated mice showed significantly higher levels of sNKG2DL than untreated mice (**Figure 4A**). Most clinically relevant, the levels of sNKG2DL were significantly higher in pediatric patients suffering from leukemia than in healthy donors (**Supplemental Figure 7**). We then analyzed the impact of sNKG2DL on NKG2D-CAR T cells. Despite NKG2D expression decreased in the presence of sMICA and sULBP2 (**Figure 4B**), the cytotoxicity of NKG2D-CAR T cells remained unaltered even when the highest doses of sNKG2DL were used (**Figure 4C**). Additionally, no differences in the MTOC polarization indexes and thus, in the formation of productive immunological synapses, were found between untreated NKG2D-CAR T cells and those treated with 500ng/ml sMICA for 72h (**Figure 4D**). In the presence of some sNKG2DL, NKG2D-CAR expression and cell proliferation affected to CD4 and CD8 T cells in a different manner (**Supplemental Figure 8**).

### 3.5 NKG2D-CAR T cells enrich the LICs compartment

**Figure 5:**
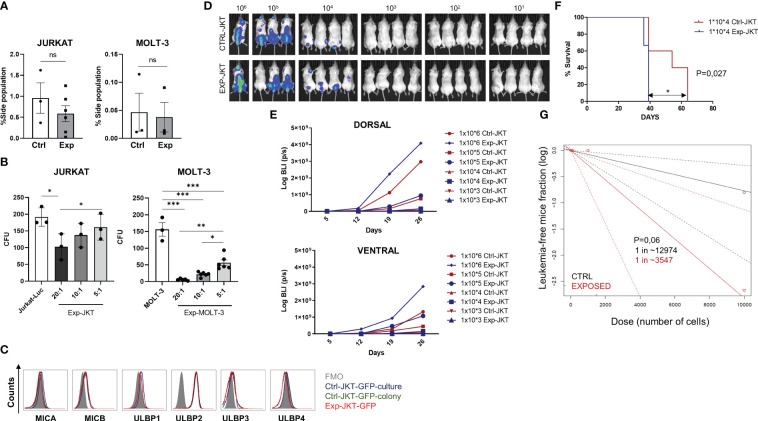
NKG2D-CAR T cells fail to target the LICs compartment. **(A)** Side population of Jurkat and MOLT-3 cells was reduced upon co-culture with NKG2D CAR T cells. Six or three different NKG2D CAR T cell donors were used for the experiments with Jurkat cells (N=6) and for MOLT-3 cells (N=3) respectively. **(B)** Colonies grown from Jurkat and MOLT-3 cells before and after co-culture with NKG2D-CAR T cells. Three replicates from three different donors were measured for each cell line (N=3). **(C)** Similar expression levels of NKG2DL were found in Jurkat-GFP-Luc cells cultured in suspension (blue histogram), Jurkat-GFP-Luc cells grown as colonies (enriched in LICs) (green histogram) and remaining Jurkat-GFP-Luc grown as colonies after co-culture with NKG2D-CAR T cells at 20:1 E:T ratio (red histogram). Kruskal-Wallis one-way ANOVA following Dunn’s *post-hoc* test was performed for multiple comparisons. Unpaired t-test was applied for two columns comparison *p < 0,05; **p < 0,01; ***p < 0,001. **(D)** Representative images of BLI measured in the mice at day+19 after infusion. **(E)** Mice engrafted with Exp-JKT and Ctrl-JKT show similar BLI. **(F)** Mice engrafted with Ctrl-JKT cells showed increased survival compared to those engrafted with Exp-JKT cells. **(G)** Exp-JKT cells showed increased ability to cause leukemia in the mice and present 3.65 fold frequency of LIC.

The inability of NKG2D-CAR T cells to eliminate the LICs could also explain the incomplete eradication of the leukemia in the *in vivo* model. To explore this possibility, we co-cultured Jurkat-GFP cells or MOLT-3 cells with NKG2D-CAR T cells for 16h (Exp-JKT/Exp-MOLT-3 cells) or left unexposed (Ctrl-JKT/Ctrl-MOLT-3) and analyzed the side population (SP). We observed a reduced percentage of SP in Exp-JKT and Exp-MOLT-3 cells compared to unexposed controls. However, this difference was not statistically significant (**Figure 5A**). The flow cytometry dot plots from the SP assays are shown in **Supplemental Figure 9**. Colony forming assays revealed that Exp-JKT and Exp-MOLT-3 cells had a significantly reduced ability to form colonies, although colonies still grew, even at high E:T ratios (**Figure 5B**); and this occurs despite the expression of NKG2DL (**Figure 5C**). To analyze if *in vivo* Exp-JKT cells had increased LIC activity, we transplanted NSG mice using different cell doses of Ctrl or Exp-JKT cells ranging from 10^6^ to 10. All mice receiving 10^5^ or 10^6^ cells developed T-ALL, although Exp-JKT groups had increased tumor burden, as measured by BLI signal (**Figures 5D, E**). For the 10^4^ dose, we found 4/7 (57%) of mice receiving Ctrl-JKT cells developed T-ALL and showed increased survival compared to mice receiving Exp-JKT, where all the 7/7 (100%) developed the disease and died by day 39 (**Figure 5F**). Mice receiving ≤10^3^ cells did not develop leukemia, regardless of the exposed/unexposed origin of the Jurkat cells (**Figure 5D**). Analysis of T-ALL engraftment revealed a 3.65 fold enriched LIC frequency in Exp-JKT cells compared with Ctrl-JKT cells and a significantly higher frequency of disease-free mice in animals transplanted with Ctrl-JKT cells (**Figure 5G**). To further explore how NKG2D-CAR T cells affected gene expression, Exp-JKT cells were sorted after co-culture and used to perform transcriptome analysis by RNAseq. We found Exp-JKT cells upregulated genes related to stemness, proliferation and survival including STAT1, Bcl-2, SMAD, LIF or L1CAM compared to unexposed (Ctrl-JKT) cells. Additionally, CD52, different gene clusters involved in immune response like IFN-γ-mediated signaling pathways, MHC class II complex, and genes related to antigen processing and presenting, T cell costimulation, or GvHD (CD86 and HLA family genes) were also upregulated (**Supplemental Figure 10A**). To further corroborate these findings, we analyzed the expression of SMAD1, LIF, L1CAM, STAT1, CD52, Bcl-2 genes by qPCR in MOLT-3 and CCRF-CEM either Ctrl or after co-culture with NKG2D CAR T cells. We found upregulation of STAT1 and L1CAM on both Exp-MOLT3 and Exp-CEM. Exp-CEM cells also showed upregulation of LIF gene (**Supplemental Figure 10B**).

## 4 Discussion

The prognosis for r/r T-ALL, is dismal and effective CAR-T cell therapeutic options are required. In this study we hypothesized that CD45RA^-^ memory T cells expressing a NKG2D-41-BB-CD3ζ CAR could serve as therapeutic approach to treat pediatric acute leukemia.

NKG2D-CAR memory T cell therapy presents several advantages over other CAR T options: 1) is based in the natural occurring NKG2D-NKG2DL tumor immunosurveillance pathway 2) NKG2D-CAR construct is fully human, avoiding immunogenicity 3) NKG2D-CAR memory T cells do not exert fratricide, facilitating CAR T manufacturing 4) spares healthy T and B cells, preventing T and B-cell aplasia (contrary to other CARTs recognizing Pan T-cell/B-cell antigens 5) CD45RA^-^ cells are less alloreactive and can be used in an allogeneic context, facilitating the access to this therapy for those patients with lymphopenia or T-cell dysfunction.

Besides heterogeneous expression of NKG2DL among different leukemia cell lines and individuals (9, 33, 34), NKG2D-CAR T cells have been shown to induce tumor elimination and long-term tumor-free survival (9, 10). For most CAR-T cell therapies, a higher expression of tumor antigen correlates with higher tumor-cell lysis. Contrary to this, we found no correlation between NKG2DL expression and NKG2D-CAR mediated cytotoxicity, and similar results have been reported for adult AML and T-ALL (9, 34) and for osteosarcoma (14, 35). It is important to note that NKG2D ligands are characterized by a variable domain structure, distinct mode of membrane anchor and diverse affinity for their receptor, and therefore, it is likely that they are not equally able to evoke activating signals in the endogenous receptor (32, 36). Since the extracellular domain of the NKG2D-CAR is the same as it is in the wild type receptor, it seems reasonable to think that although the presence of NKG2DL is needed to mediate NKG2D-CAR T cells cytotoxicity, the binding affinity of NKG2D-CAR to different ligands and how they induce the downstream activating signals, may vary among the different NKG2DL. Moreover, in addition to their differences in binding affinity, the NKG2DL may have different ability to induce NKG2D-CAR T cells activation. Additionally, tumor cells may present intrinsic resistance mechanisms to inhibit CAR T cells activation and killing. All these factors could account for the absence of correlation between NKG2DL expression and NKG2D CAR cytotoxicity that we and others have observed.

In our *in vitro* experiments, AML cells showed the highest sensitivity to NKG2D-CAR T cells’ lysis. However, the efficacy of NKG2D CAR T cells in patients suffering from AML has been already shown in a clinical trial (10). Since we observed NKG2D CAR T cells were also effective against T-ALL (especially against Jurkat cells) and there are still no effective CAR T cell therapies available to treat this disease, we set the focus in exploring the potential use of NKG2D CAR T cells as treatment for T-ALL using Jurkat cells. *In vivo*, weekly doses of NKG2D-CAR T cells improved treatment efficacy compared to the single CAR T cells dose. However, despite the persistence of CAR-T cells in the bone marrow and their ability to degranulate in response to leukemic blasts, even the multiple doses regimen failed to completely eradicate the tumor and the mice died from leukemia progression only one week after the last CAR T cells infusion. Importantly, the persistence of those CAR T cells infused once and in the multiple doses regimen could not be compared, since the animals were euthanized at different time points after the infusion, following humane endpoint criteria, when they presented symptoms of leukemia progression (32 days for the single dose and just 4 days after the last infusion in the multiple doses group). Another limitation of our *in vivo* model could rely on the use of Jurkat cells to establish the T-ALL, as cell lines accumulate mutational burden and may have a more aggressive behavior than primary tumor cells (37, 38). The high proliferation rate of Jurkat cells along with the limited *in vivo* expansion of CD45RA^-^ cells may cause an inversion of the E:T ratios, rendering the CAR T cells unable to eliminate a massive number of tumor cells. It is important to note that, *in vitro*, we did not observe differences in the cytotoxic capacity of NKG2D-CAR T cells against leukemia cells regardless if they were obtained from CD45RA^-^ cells or total PBMC. However, these experiments are limited to 4 hours, and the different composition in naïve, effector memory and central memory T cells of PBMC and CD45RA^-^ cells, could lead to a different behavior *in vivo*. Additionally, the inability of NKG2D-CAR T cells to cure the animals could be related with immunosuppressive mechanisms developed by the leukemic cells. Release of sNKG2DL by tumor cells is a well-known immune evasion mechanism (22, 33). Mice engrafted with Jurkat cells showed increased levels of sNKG2DL, compared to healthy controls. Moreover, the highest levels of sNKG2DL were measured in those mice treated with NKG2D-CAR T cells. NKG2D-CAR T CD45RA^-^ cells have no surface expression of NKG2DL (15), which prevents fratricide and makes the shedding of sNKG2DL unlikely. Tumor cell lysis could also account for the increased levels of sNKG2DL in the treated mice as well, and similar observations have been made in pediatric patients suffering from r/r leukemia after receiving NKG2D-CAR T cells (39). Furthermore, Jurkat cells may secrete sNKG2DL as a counter-attack against NKG2D-CAR T cells, and according to this, after CAR exposure, Jurkat cells upregulated genes involved in immune response, including HLA-E that could inhibit NK cell tumor-immunosurveillance through ligation to NKG2A receptor.

The suppressive effects of sNKG2DL on NKG2D receptor have been reported (22, 40–42). However, the impact of sNKG2DL on NKG2D-CAR T cells has been studied at a lesser extent (43). *In vitro*, we found only elevated concentrations of sNKG2DL (500ng/ml) altered NKG2D expression, but not sufficiently to impair cytotoxicity. In the mice, the levels of sNKG2DL were far below 10ng/ml. Since a concentration of sNKG2DL of 500ng/ml was not enough to reduce NKG2D CAR T cells´ cytotoxicity *in vitro*, we hypothesize that the low concentration of sNKG2DL found in the animals (10 ng/ml) was not sufficient to cause the treatment failure in the *in vivo* model. Moreover, the levels of sNKG2DL present in the serum of patients would be insufficient to impair NKG2D-CAR T cells functionality. A different impact of sNKG2DL on the chimeric and the endogenous NKG2D receptors had already been reported and could be related to the different signaling pathways in both receptors (43).

Inability of NKG2D-CAR T cells to eliminate LICs could also explain the lack of a robust response in the murine model. It has been described that AML LICs downregulate NKG2DL expression, and show enhanced resistance to NK cell-mediated killing (44), however, we observed that after co-culture with NKG2D-CAR T cells, Exp-JKT had similar NKG2DL expression than Ctrl-JKT. Therefore, immunoescape of Jurkat LICs from NKG2D-CAR Ts may not be attributed to a downregulation of NKG2DL.

After co-culture with NKG2D-CAR T cells, Jurkat cells showed upregulation of genes involved in proliferation, survival, migration and stemness. *In vivo*, these cells demonstrated increased ability to cause leukemia, indicating NKG2D-CAR T cells failed to completely eliminate LICs, and thus, they could be responsible for treatment inefficacy. Further experiments using CCRF-CEM and MOLT-3 T-ALL leukemia cell lines, corroborated upregulation of STAT-1 and L1CAM after treatment with NKG2D-CAR T cells. LIF gene was also upregulated in CCRF-CEM cells. STAT1 has been reported to be a promoter of leukemia development and STAT1 deficient mice have been shown to be partially protected from leukemia development (45). Moreover, it has also been described that in the absence of STAT-1, the expansion potential of LICs is severely reduced (46). L1CAM positive cells have been shown to possess a higher tumorigenic potential *in vitro* and *in vivo*, and a chemoresistant phenotype. Additionally, elevated L1CAM expression has been reported to confer cancer stem cell properties in a variety of tumors and has been associated with poorer outcomes in many cancer types (47). Currently, it is known that LIF acts regulating cell proliferation, differentiation and survival, as well as maintaining the state of pluripotency and self-renewal of stem cell populations (48). All these data taken together, suggest that after NKG2D-CAR T cells treatment, leukemic cells are enriched in LICs.

Overall, we provide preclinical evidence of the anti-tumor activity of NKG2D-CAR T cells against pediatric acute leukemia. Based on our results, the use of NKG2D-CAR T cells as single agent may not be sufficient to cure the patients. Instead, we propose using NKG2D-CAR therapy in combination with other therapeutic approaches to specifically target LICs. Different druggable genes including Bcl2, STAT1, and CD52 were upregulated in remaining LICs after NKG2D-CAR treatment. Bcl2 and STAT1 are downstream targets of JAK/STAT pathway; therefore, the use of JAK/STAT inhibitors such as Ruxolitinib could be a useful agent for a combinatorial therapy. Alternatively, the Bcl2 inhibitor Venetoclax or the anti-CD52 specific antibody Alemtuzumab could also be part of the combinatorial therapy. The use of NKG2D-CAR T cells as bridge to HSCT or as adoptive cell therapy after HSCT could also be a therapeutic option. In this regard there are early signs that subsequent transplantation of patients who have achieved remission with CAR-T may be a potentially viable and successful strategy (18, 49, 50). Importantly, combined therapy should be tailored to each patient to ensure specificity and avoid toxicity, and therefore, further studies to explore the safety and efficacy of these combinatorial approaches using NKG2D-CAR T cells are warranted.

## Data availability statement

The datasets presented in this study can be found in online repositories. The names of the repository/repositories and accession number(s) can be found below: GEO with the accession number GSE228528.

## Ethics statement

All patients or their guardians gave their written informed consent for participation in this study in accordance with the Helsinki protocol. The studies were performed according to the guidelines of the local Ethics Committee (PI-3374). The studies were conducted in accordance with the local legislation and institutional requirements. The animal study was approved by the Spanish National Cancer Research Centre (CNIO) Animal Care and Use Committee and by the ethics committee of the Instituto de Salud Carlos III (ISCIII) and the CAM (PROEX173/17). The study was conducted in accordance with the local legislation and institutional requirements.

## Author contributions

AF conceived the study, performed experiments, analyzed data, and wrote the paper. AE, MI-N, GE, CC-S, AL, BC, CR-A, AN-Z, CM-D, MB-P, AO, MV-G and CF performed experiments and analyzed data. JM and MV-G provided facilities and supervised the study. AP-M conceived and financially supported this study. LF conceived the study, designed and performed experiments, analyzed data and wrote the paper. All authors contributed to the article and approved the submitted version.
