# Supplementary material for: NKG2D-CAR memory T cells target pediatric T-cell acute lymphoblastic leukemia in vitro and in vivo but fail to eliminate leukemia initiating cells
Source: Front Immunol. 2023 Oct 18;14:1187665. doi: 10.3389/fimmu.2023.1187665 (PMC10622787; doi:10.3389/fimmu.2023.1187665)
Supplement: Supplementary file 1 [file DataSheet_1.pdf]

Supplementary Material

NKG2D-CAR memory T cells target pediatric T-cell acute lymphoblastic leukemia in vitro and in vivo but fail to eliminate leukemia initiating cells

Marta Ibáñez-Navarro†[1], Adrián Fernández†[1], Adela Escudero[2], Gloria Esteso[3], Carmen Campos-Silva[3], Miguel Ángel Navarro-Aguadero[1], Alejandra Leivas[1,4], Beatriz Ruz Caracuel[2], Carlos Rodríguez-Antolín[5,6], Alejandra Ortiz[1,4], Alfonso Navarro-Zapata[7], Carmen Mestre-Durán[7], Manuel Izquierdo[8], María Balaguer-Pérez[1,9], Cristina Ferreras[7], Joaquín Martínez[1,4], Mar Valés-Gómez[3], Antonio Pérez-Martínez*[7,10,11], Lucía Fernández*[1].

*** Correspondence:**

Lucía Fernández: lvfernandez@cnio.es

Antonio Pérez-Martínez: aperezmartinez@salud.madrid.org

†MIN and AF contributed equally as first co-authors

*LF and APM contributed equally as senior and corresponding authors

Supplemental Table 1

Reactivity	Antigen/ dye	Clone	Flurochrome	Reference
**Human**	MICA	159227	PE	R&D systems, MN, USA, FAB1300P
**Human**	MICB	236511	APC	R&D systems, FAB1599A
**Human**	ULBP-1	170818	PE	R&D systems, FAB1380P
**Human**	ULBP-2,5,6	165903	APC	R&D systems, FAB1298A
**Human**	ULBP-3	166510	PE	R&D systems, FAB1517P
**Human**	ULBP-4	709116	APC	R&D systems, FAB6285A
**Human**	CD45RA	HI100	APC	Biolegend, CA, USA, 304111
**Human**	NKG2D	1D11	PE	Biolegend, 320806
**Human**	NKG2D	1D11	BV421	BD Pharmingen 743558
**Mouse**	CD45	30F11	APC	Biolegend, 103112
**Human**	CD45	2D1	PerCP-Cy5.5	Biolegend, 368504
**Human**	CD45	HI30	FITC	BD Biosciences, 555482
**Human**	CD3	HIT3a	PE-Cy7	Biolegend, 300316
**Human**	CD3	SP34-2	PerCP-Cy5.5	BD Biosciences
**Human**	CD3	OKT3	FITC	Biolegend, 317306
**Human**	CD8	SK1	FITC	Biolegend, 344703
**Human**	CD8	SK1	APC-Cy7	BD Pharmingen 557834
**Human**	CD8	SK1	PE-Cy7	Biolegend, 344712
**Human**	CD4	OKT4	APC-Cy7	Biolegend, 317418
**Human**	CD4	OKT4	PerCP	Biolegend, 31743125
**Human**	CD4	OKT4	AF 700	Biolegend 317426
**Human**	CD4	OKT4	PerCP-Cy5.5	Biolegend, 317428
**Human**	CD107a	REA792	APC	Miltenyi Biotec, 130-111-847
**Human**	PD-1	EH12.1	PE-CF594	BD Horizon, 565024
**Human**	TIM-3	F38-2E2	PE-Vio770	Miltenyi Biotec, 130-121-334
**Human**	IFN-g	REA700	PE	Miltenyi Biotec, 130-097-940
**Human**	IL-2	REA689	PE	Miltenyi Biotec, 130-129-245
**Human**	TNFa	MAb11	APC	BD Pharmingen, 562084
**Human**	CD25	BC96	APC	Biolegend, 302610
**Human**	CD62L	DREG-56	FITC	BD Pharmingen, 555543
**Human**	IgG Fc		PE	Thermo Fisher 12-4998-82
**-**	DAPI	-	-	Sigma-Aldrich, D8417
**-**	7AAD	-	-	BD Biosciences, 559925
**-**	CellTrace Violet™	-	-	Thermo Fisher, C34557
**-**	Aqua	-	-	ThermoFisher, L34957

Supplemental Table 1. Fluorochrome-labeled monoclonal antibodies used for FCM analysis

Supplemental Table 2

Antibody/ protein	Reference
**COATING ANTIBODIES**	
**MICA**	R&D systems, MAB1300
**MICB**	R&D systems, AF1599
**ULBP-1**	R&D systems, AF1380
**ULBP2**	R&D systems, AF1298
**ULBP-3**	R&D systems, AF1517
**ULBP-4**	R&D systems, MAB6285
**RECOMBINANT PROTEINS**	
**rhMICA**	R&D systems, 1300-MA
**rhMICB**	R&D systems, 1599-MB
**rhULBP-1**	R&D systems, 1380-UL
**rhULBP-2**	R&D systems, 1298-UL
**rhULBP-3**	R&D systems, 1517-UL
**rhULBP-4**	R&D systems, 6285-UL
**rhNKG2D Fc Chimera**	R&D systems 1299NK
**SECONDARY ANTIBODIES**	
**Biotin-MICA**	R&D systems, BAF-1300
**Biotin-MICB**	R&D systems, BAF1599
**Biotin-ULBP-1**	R&D systems, BAF1380
**Biotin-ULBP2**	R&D systems, BAF1298
**Biotin-ULBP-3**	R&D systems, BAF1517
**Goat-anti-ULBP-4**	R&D systems, AF6285
**DETECTION ANTIBODIES**	
**HRP-straptavidin**	Biolegend, 405210
**HRP-anti-goat**	Invitrogen, A16005

Supplemental Table 2. NKG2DL recombinant human proteins and HRP antibodies for ELISA and impact of sNKG2DL in NKG2D CAR T cells.

Supplemental Figure 1

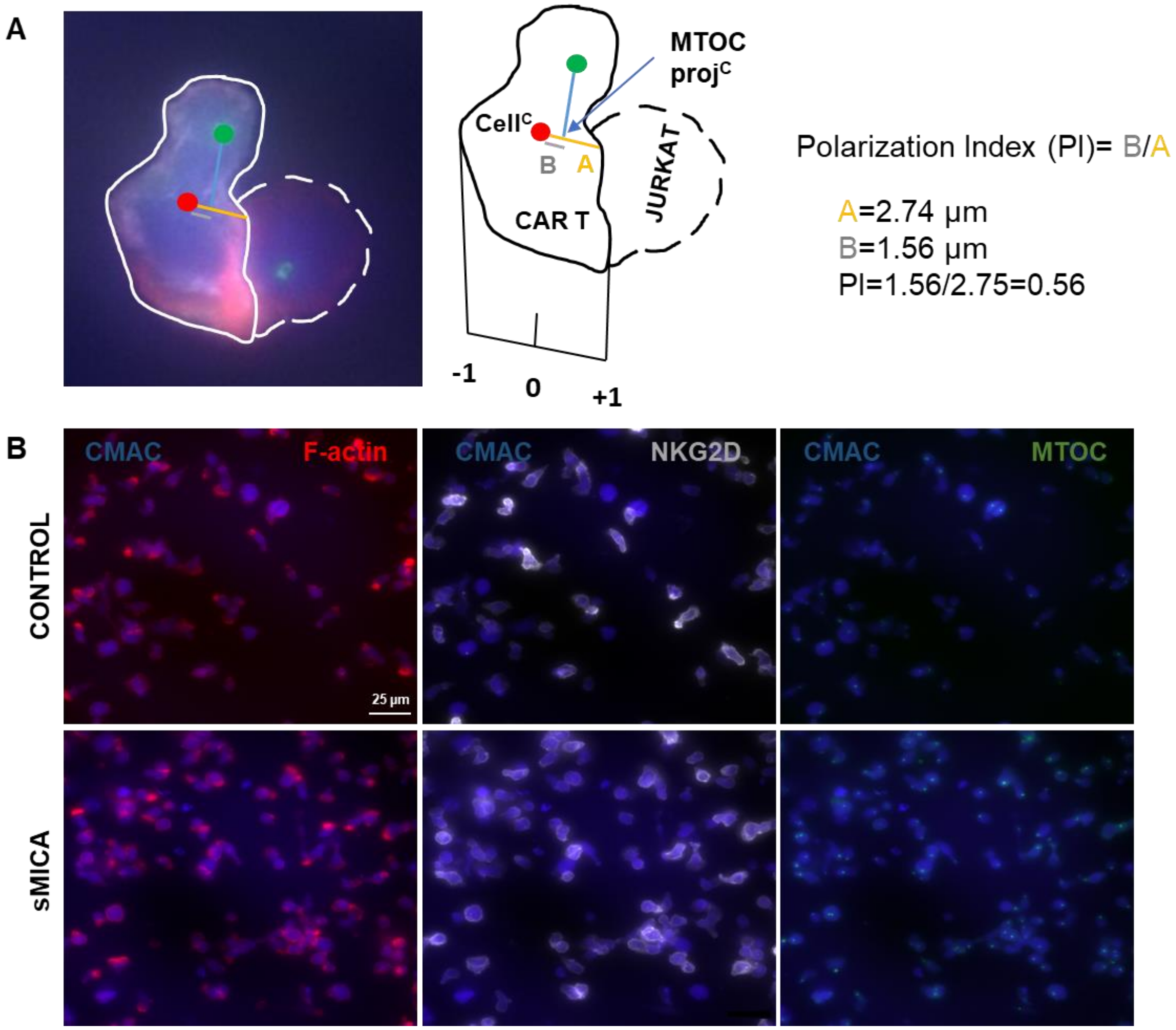

Supplemental Figure 1. **Quantification of polarized Immune synapses.** A) MTOC polarization index (MTOC PI) was calculated by measuring the distance of the cell geometric center (Cell^c) (A distance) to the immune synapse, and the distance between the projection of the MTOC center of mass (MTOC^c) on the vector defined by the Cell^c–synapse axis to the Cell^c (B distance). Cell^c position was taken as origin to measure distances, and those values in opposite direction to the synapse were taken as negative. MTOC PI were calculated, as described, as the ratio of distances A and B (MTOC PI=B/A) , ranging from +1 to -1. Therefore, MTOC PI values were normalized by cell size and shape as previously described (Herranz, *et al. Frontiers Immunology*, 2019). B) Representative epifluorescence images of immunological synapses of CMAC labeled Jurkat cells (blue) challenged for 30 min with NKG2D CAR T cells untreated or after treatment with 500ng/ml sMICA for 72h at 1:1 E:T ratio. F-actin, NKG2D and MTOC are labeled in red, gray and green respectively.

Primers for qPCR analysis	
hSTAT1_qPCR_Fw	5'-ATGGCAGTCTGGCGGCTGAATT-3'
hSTAT1_qPCR_Rv	5'-CCAAACCAGGCTGGCACAATTG-3'
hSMAD_qPCR_Fw	5'-TTGGCACAGTCTGTGAACCATGG-3'
hSMAD_qPCR_Rv	5'-GTAACATCCTGGCGGTGGTATTC-3'
hBCL-2_qPCR_Fw	5'-ATCGCCCTGTGGATGACTGAGT-3'
hBCL-2_qPCR_Rv	5'-GCCAGGAGAAATCAAACAGAGGC-3'
hLIF_qPCR_Fw	5'-AGATCAGGAGCCAACTGGCACA-3'
hLIF_qPCR_Rv	5'-GCCACATAGCTTGTCCAGGTTG-3'
hCD52_qPCR_Fw	5'-CTCTCAGGACAAAACGACACCAG-3'
hCD52_qPCR_Rv	5'-CTAAGGCTGAGACGTGTCACCT-3'
hL1CAM_qPCR_Fw	5'-TCGCCCTATGTCCACTACACCT-3'
hL1CAM_qPCR_Rv	5'-ATCCACAGGGTTCTTCTCTGGG-3'

Supplemental Table 3. Primers used for qPCR analysis of STAT1, SMAD, Bcl-2, LIF, CD52 and L1CAM.

Supplemental Figure 2

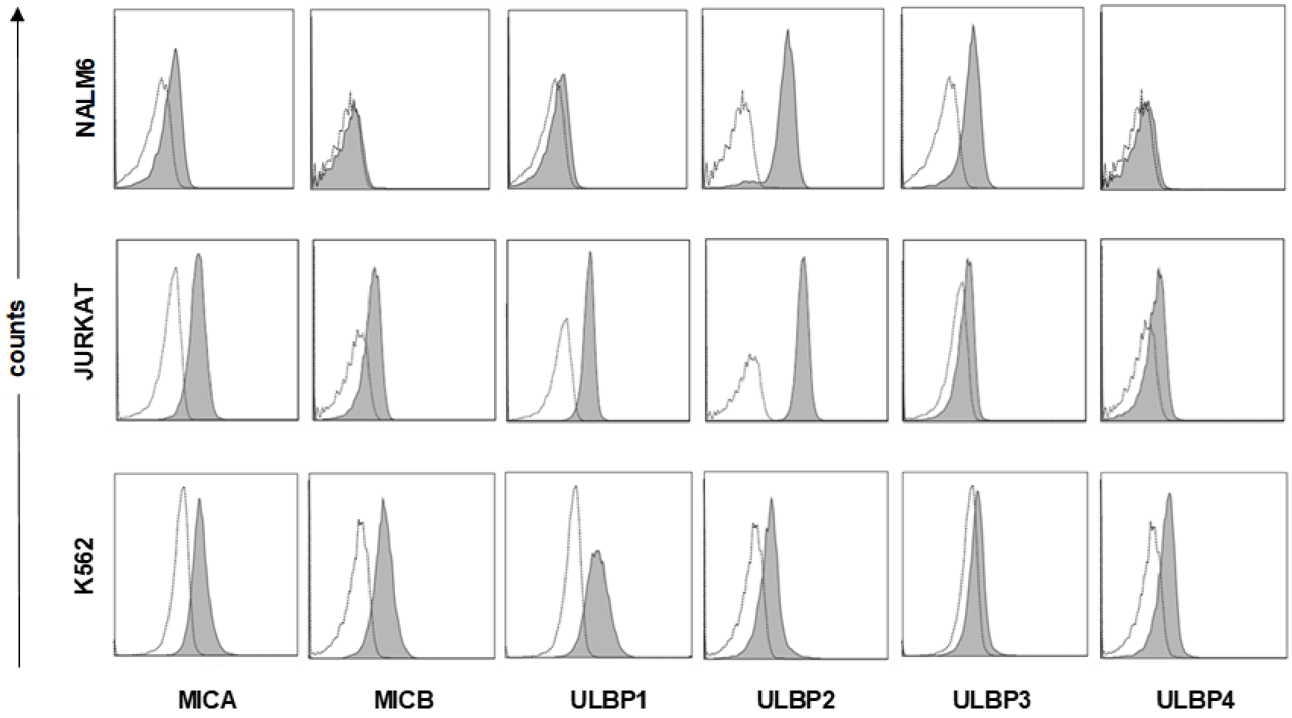

Supplemental Figure 2. Representative histograms showing NKG2DL expression of different leukemia cell lines by FCM. Filled histograms represent stained samples while blank histograms are related to unstained cells.

Supplemental Figure 3

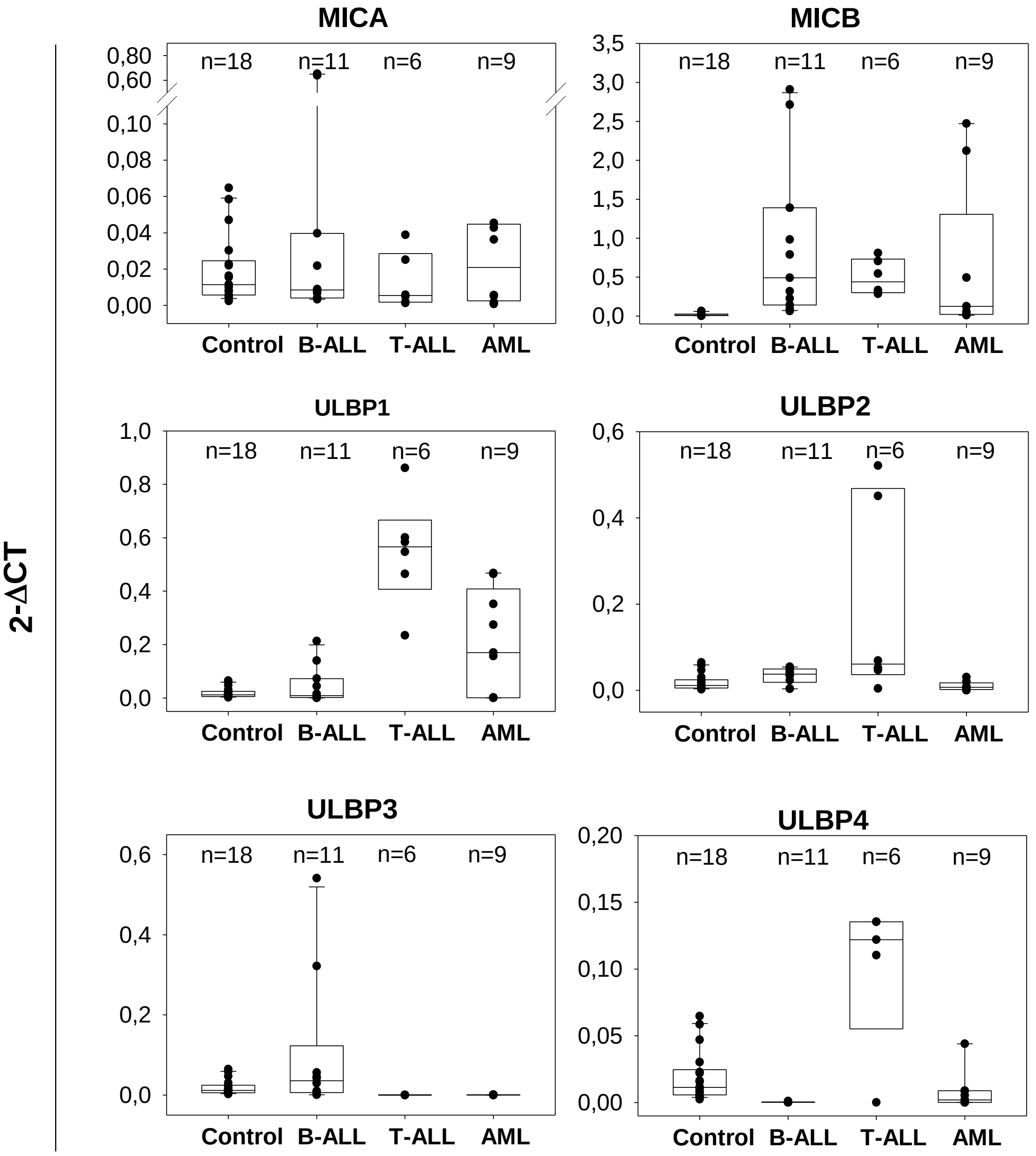

Supplemental Figure 3. mRNA levels of NKG2DL on leukemia cell lines. B-ALL (NALM-6, REH, RS4;11, SEM and TOM-1), T-ALL (CEM, JURKAT and MOLT-3), AML(ME-1, K562, KASUMI, MV4;11). N corresponds to duplicated or triplicated experiments for each cell line. PBMCs from healthy donors were used as control.

Supplemental Figure 4

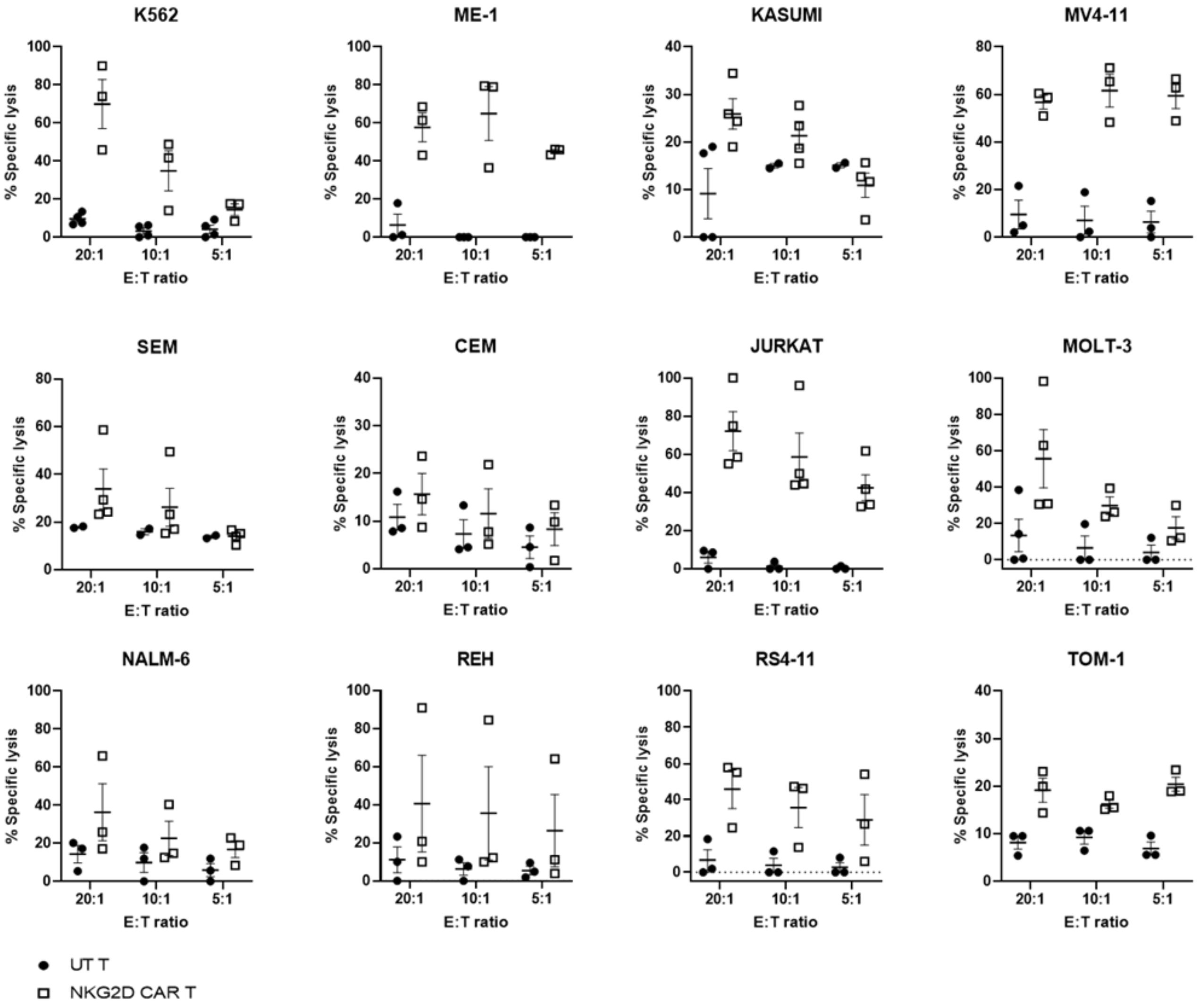

Supplemental Figure 4. Cytotoxicity of NKG2D CAR T cells against leukemia cell lines at different E:T ratios.

Supplemental Figure 5

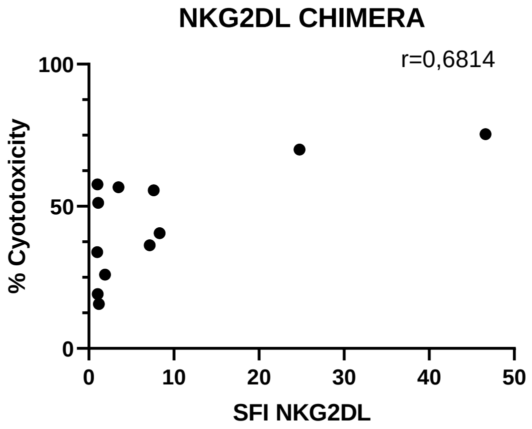

Supplemental Figure 5. Correlation analysis between NKG2DL expression of leukemia cell lines and their susceptibility to NKG2D-CART cytotoxicity. Collective expression of NKG2DL was analyzed by using rhNKG2D Fc chimera and Goat anti-human IgG-PE antibody (details can be found at ST2 and ST1). Parametric Pearson correlation analysis was applied between X and Y values.

Supplemental Figure 6

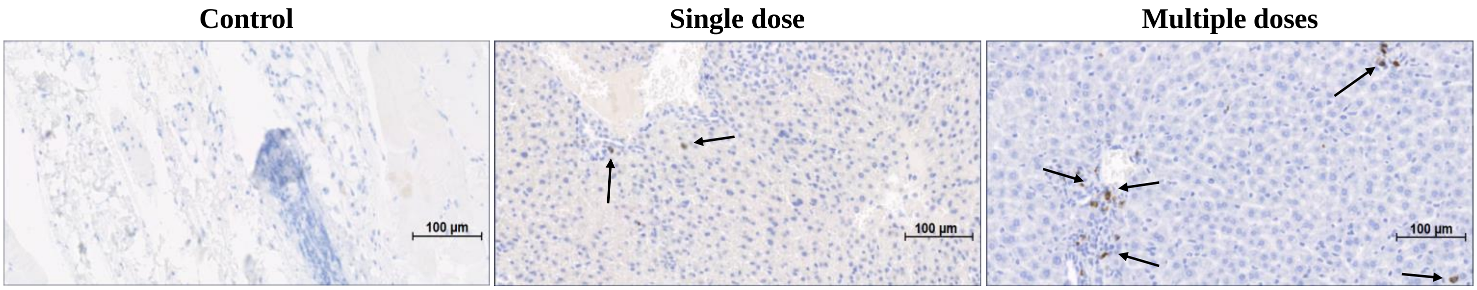

Supplemental Figure 6. IHC staining of BM sections of mice remained untreated (control), treated with single dose of NKG2D-CART (sample collected 32 days post CAR T cells infusion) or with multiple doses of NKG2D-CART (sample collected 4 days post CART cells infusion). Brown color correspond to granzyme B staining (arrows), which associates with the presence of degranulating NKG2D-CART cells in the sample.

Supplemental Figure 7

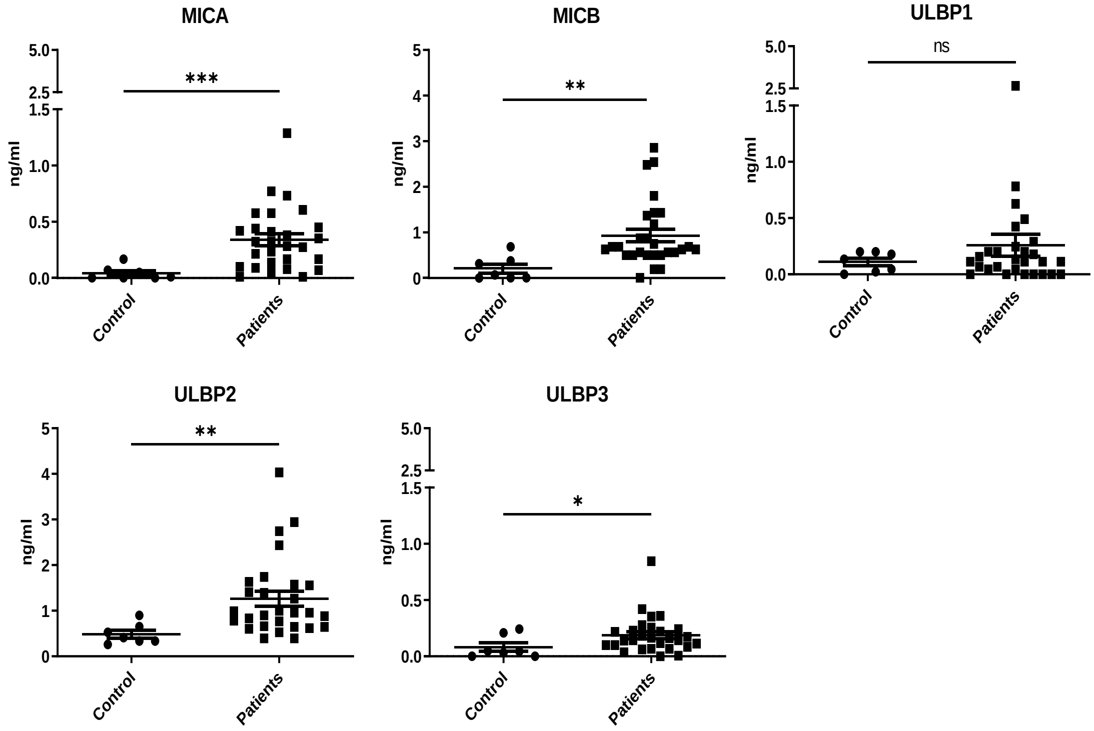

Supplemental Figure 7. Levels of sNKG2DL measured by ELISA in the serum of pediatric patients suffering from leukemia (N=28; B-ALL N=20, AML N=7, T-ALL N=1) at diagnosis, and in healthy donors (N=7).

Supplemental Figure 8

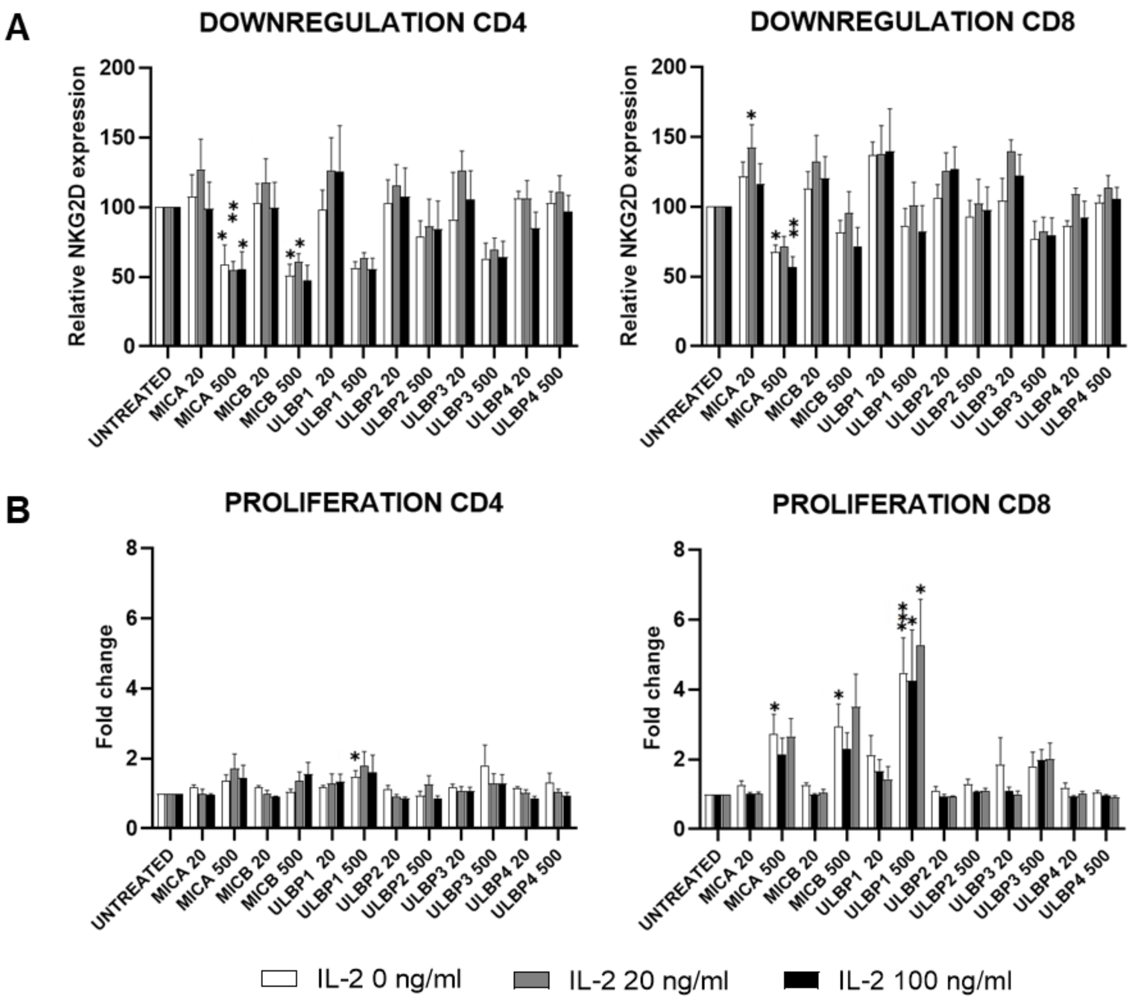

Supplemental Figure 8. Impact of sNKG2DL on NKG2D-CAR T cells. A) Relative expression of NKG2D receptor in CD4 and CD8 subsets. B) Fold change proliferation in CD4 and CD8 subsets. Friedman one-way ANOVA following Dunn's post-hoc test was performed. *p < 0,05; **p < 0,01; ***p < 0,001. Two-way ANOVA following Tukey's post-hoc test was performed. *p < 0,05; **p < 0,01; ***p < 0,001

Supplemental Figure 9

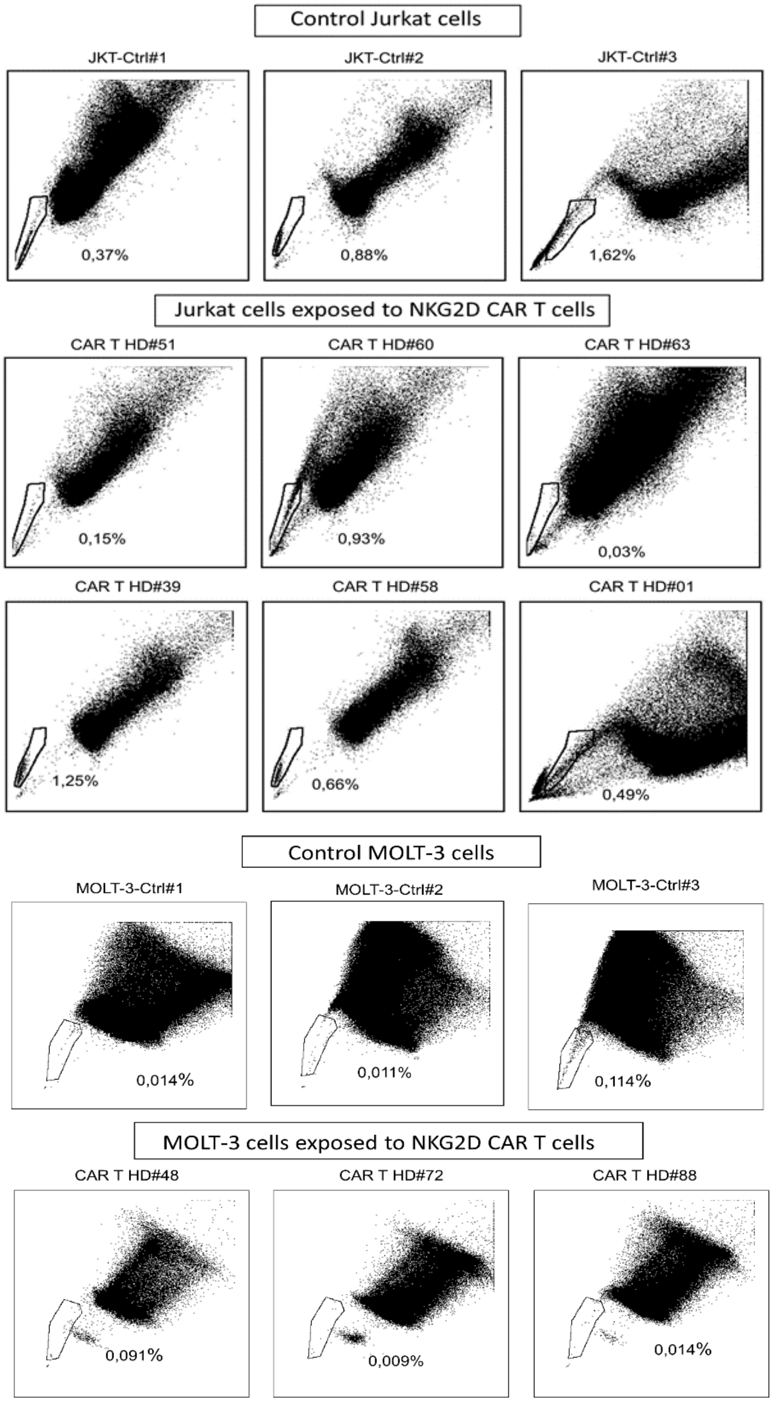

Supplemental Figure 9. Flow cytometry dot plots from the side population experiments. The percentage of side population was analyzed in unexposed Jurkat and MOLT-3 cells (Ctrl) (N=3) and in Jurkat and MOLT-3 cells after co-culture with NKG2D-CAR T cells from different donors (N=6 for Exp-JKT, and N=3 for Exp-MOLT-3).

Supplemental Figure 10

A

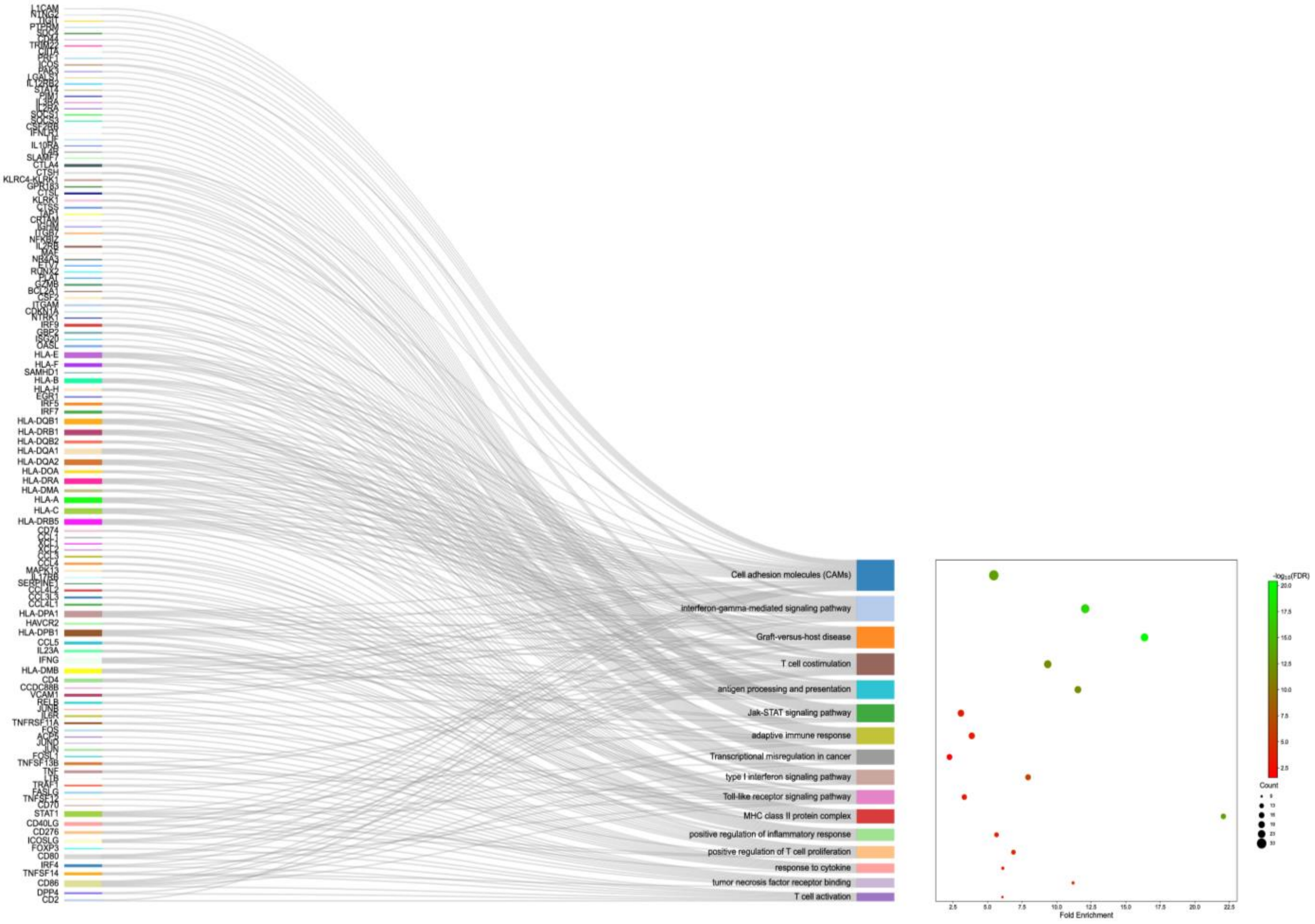

B

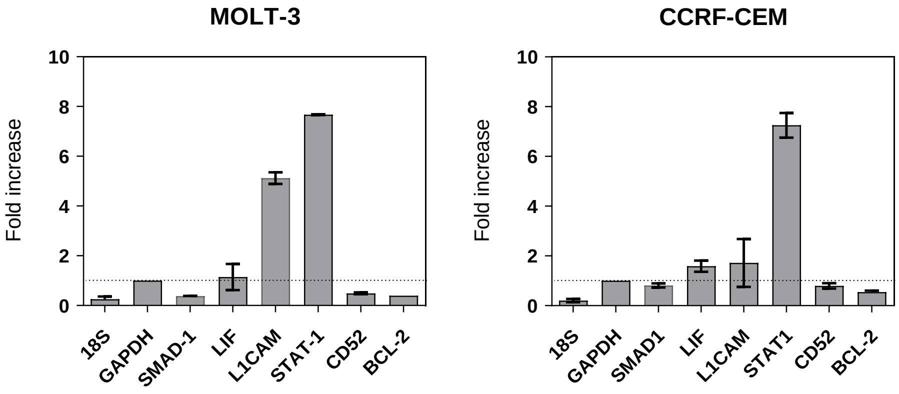

Supplemental Figure 10. A) Differential gene expression in Jurkat LICs upon NKG2D-CAR pressure. RNAseq heat map showing upregulation of genes involved in proliferation, survival, and immune response, including STAT1, Bcl-2, SMAD, LIF or L1CAM, in Jurkat cells after co-culture with NKG2D CAR T cells, B) After co-culture with NKG2D-CAR T cells, Exp-MOLT-3 showed upregulation of STAT1 and L1CAM. Exp-CEM cells showed upregulation of STAT1, L1CAM and LIF genes by qPCR analysis. The graph shows mean±SEM from two independent experiments performed in triplicates (N=2).
